# *Bacillus aerophilus* A5 suppresses tobacco root rot and gene expressions involved in fungal chitin metabolism

**DOI:** 10.1080/15592324.2026.2723413

**Published:** 2026-09-28

**Authors:** Xinrui Li, Fei Xiong, Ning Jiang, Tianfu Zhao, Yanxia Hu, Yongzhan Cai, Yuanhu Xuan, Xiaotong Gai

**Affiliations:** a Yunnan Academy of Tobacco Agricultural Sciences, Kunming, People's Republic of China; b Department of Chemical Biology, State Key Laboratory of Elemento-Organic Chemistry, National Pesticide Engineering Research Center (Tianjin), Nankai University, Tianjin, People's Republic of China; c Dali Tobacco Company of Yunnan Dali, Dali, People's Republic of China; d Qujing Tobacco Company of Yunnan Qujing, Qujing, People's Republic of China

**Keywords:** Tobacco root rot, Bacillus aerophilus, Fusarium, biological control, UDP-N-acetylglucosamine, cell wall precursor metabolism

## Abstract

Tobacco root rot is a destructive soil-borne disease that constrains sustainable tobacco production in Yunnan. *Fusarium oxysporum* and *Fusarium solani* species complex are currently considered the principal pathogens associated with this disease. Therefore, effective biocontrol agents are needed, and understanding their pathogen-side modes of action is essential for improving biological management strategies. This study screened 100 bacterial strains isolated from the tobacco rhizosphere using plate confrontation assays and identified A5 as a broad-spectrum antagonistic strain. A5 consistently inhibited four tobacco root rot-associated *Fusarium* species, with hyphal growth inhibition rates of 63.31%, 59.42%, 69.64%, and 68.82%, respectively. Phylogenetic analyses of 16S rRNA and *gyrB* sequences placed A5 close to *Bacillus aerophilus*, supporting its classification as a *B. aerophilus*-related biocontrol candidate. To investigate the fungal response to A5, *F. solani* was exposed to 10% sterile A5 fermentation supernatant and analyzed using transcriptomic sequencing. GO and KEGG enrichment analyses showed differentially expressed genes (DEGs) involved in UDP-*N*-acetylglucosamine biosynthesis and associated cell wall precursor metabolic processes. Because *F. oxysporum* has a more established genetic manipulation system in tobacco root rot research, this species was used for downstream functional validation. RT-qPCR confirmed the significant expression responses of FOXG_19023, FOXG_10443, and FOXG_12345 after the treatment with A5 supernatant. Subsequent construction of knockout mutants demonstrated that deletion of FOXG_19023 significantly restricted colony growth, suggesting that FOXG_19023 may contribute to vegetative growth of *F. oxysporum*, although its specific role in UDP-*N*-acetylglucosamine-related cell wall metabolism remains to be explored. Overall, A5 was identified as a tobacco root rot biocontrol bacterium with activity against *Fusarium* pathogens. These results further suggest that A5 may affect expression of genes involved in pathogen UDP-*N*-acetylglucosamine-related metabolism, and this response may be associated with impaired *Fusarium* hyphal growth. This study provides a potential biocontrol resource for sustainable tobacco root rot management and offers candidate pathogen-side targets for future studies on the molecular mechanisms underlying *Bacillus*-mediated suppression of *Fusarium*.

## Background

Tobacco (*Nicotiana tabacum* L.) is an economically important crop worldwide; however, its sustainable production is increasingly constrained by soil-borne diseases.^1^ Fusarium root rot is among the most damaging root diseases in major tobacco-producing areas.^2–7^ In China, this disease has become particularly prevalent in the southwestern tobacco-growing regions, including Yunnan Province. *Fusarium oxysporum* and members of the *Fusarium solani* species complex are frequently detected as the dominant causal agents.^8–10^ Qujing is the largest tobacco-growing region in Yunnan Province and is also the area most severely affected by root rot, with a disease incidence of up to 32.1%. In this region, *F. oxysporum* is the primary pathogen causing tobacco root rot.^11–13^ In the Lincang growing region of southwestern China, tobacco root rot has been attributed to *F. solani.* ^6^ ^,^ ^14^ These pathogens invade tobacco roots and vascular tissues, causing root decay, impaired water and nutrient uptake, reduced plant vigor and plant death under severe disease pressure.^8–10^ Their survival in soil as mycelia or chlamydospores further complicates disease management, especially once Fusarium root rot has become established in the field.^15^ Chemical fungicides, including carbendazim, thiophanate-methyl, and other benzimidazole fungicides, remain widely used for tobacco root rot management.^16^ Although these fungicides can suppress diseases in the short term, repeated application may promote resistant pathogen populations, disrupt beneficial soil microorganisms, and increase pesticide residues in agricultural ecosystems.^17^ ^,^ ^18^ These concerns have strengthened the need for biological control as a more sustainable approach to soil-borne disease management. Compared with chemical control, microbial biocontrol can directly antagonize pathogens and reshape beneficial interactions in the rhizosphere.^19^ ^,^ ^20^

Root rot occurs at the root–soil interface, where disease development is shaped by interactions among host roots, pathogenic *Fusarium* populations, and beneficial rhizosphere microorganisms.^8^ ^,^ ^21^ The plant-associated microbiome is increasingly viewed as an extension of plant immunity.^22^ Rhizosphere-colonizing beneficial microorganisms can promote plant growth, improve nutrient acquisition, increase stress tolerance, and activate host defense responses.^23–27^ During pathogen invasion, the rhizosphere microbiome often shifts markedly, and taxa associated with disease suppression may become enriched. These microorganisms can limit pathogen development through niche competition, antimicrobial activity, community modulation, and induction of systemic resistance.^28–32^ Within this group, *Bacillus* spp. are among the most widely studied and practically used biocontrol agents because they produce stress-resistant endospores, form biofilms, colonize plant roots, and synthesize diverse antimicrobial compounds.^33^ ^,^ ^34^ Their antagonistic activity is closely linked to the production of extracellular antifungal metabolites. Numerous *Bacillus* strains contain rich biosynthetic gene clusters, including non-ribosomal peptide synthetases and polyketide synthases, which mediate the production of lipopeptides, polyketides, antimicrobial peptides, and other specialized metabolites.^35–37^ In addition, *Bacillus* spp. can produce volatile organic compounds and cell wall-degrading enzymes, including chitinases and *β*-1,3-glucanases.^38^ ^,^ ^39^ These extracellular factors may suppress fungal growth by altering hyphal morphology, damaging membrane or cell wall integrity, inducing oxidative stress or interfering with fungal development.^39–41^ Nevertheless, current studies on *Bacillus*-mediated biocontrol have mainly emphasized strain screening, disease control performance, and metabolite identification. The molecular processes affected by the pathogen remain poorly understood, especially in *Fusarium* species associated with tobacco root rot.

The fungal cell wall offers a relevant target framework for investigating these pathogen-side processes. This structure is dynamic and tightly regulated, allowing fungi to maintain their cell shape, resist turgor pressure, support polarized hyphal growth, and withstand environmental and host-derived stresses.^42^ In most fungi, the conserved inner wall skeleton consists mainly of chitin and *β*-glucans, whereas outer wall polymers and glycosylated proteins contribute to surface properties, including hydrophobicity, adhesion, and immune recognition.^43^ ^,^ ^44^ As a *β*-1,4-linked homopolymer of *N*-acetylglucosamine, chitin is a major determinant of fungal cell wall rigidity and mechanical strength.^43^ ^,^ ^45^ UDP-*N*-acetylglucosamine is the directly activated sugar donor used during chitin synthase-catalyzed chain elongation and is a key metabolic substrate for fungal chitin biosynthesis.^46–48^ Structural studies have shown that chitin synthase transfers GlcNAc from UDP-GlcNAc to the growing chitin chain and translocates the nascent polymer across the plasma membrane.^47^ These observations support chitin biosynthesis and precursor supply as important targets for antifungal intervention.^45^ ^,^ ^47^ ^,^ ^49^ However, it remains unclear whether extracellular factors produced by *Bacillus* suppress tobacco root rot-associated *Fusarium* by disturbing UDP-GlcNAc-associated cell wall precursor metabolism.

This study screened 100 bacterial isolates for their antagonistic activity against *Fusarium* pathogens associated with tobacco root rot. Strain A5 was identified as a *Bacillus aerophilus*-related biocontrol candidate based on 16S rRNA and *gyrB* sequence analyzes. A5 exhibited broad-spectrum inhibitory activity against four tobacco root rot-associated *Fusarium* isolates. To examine the pathogen-side basis of this antagonism, we analyzed the transcriptomic response of *Fusarium* to the A5 cell-free fermentation supernatant, focusing on pathways associated with UDP-GlcNAc biosynthesis and fungal cell wall homeostasis. Subsequently, candidate genes were validated using RT-qPCR and functional analysis in *F. oxysporum*. These findings provide insights into the molecular basis of *Bacillus*-mediated suppression of tobacco *Fusarium* root rot and identify potential fungal targets related to cell wall precursor metabolism.

## Materials and methods

### Survey of the severity of tobacco root rot and soil sample collection

Field sampling was conducted in tobacco-growing areas of Yuxi, Yunnan Province, China. The local soil type was red soil, and the cultivated tobacco variety was ‘Yunyan 87’. The soil samples were grouped according to field root rot severity into four categories: non-infected (S_0), mildly infected (S_L), moderately infected (S_M), and severely infected (S_H). Disease incidence was defined as 0% for S_0, < 10% for S_L, 10%–50% for S_M, and > 50% for S_H. Rhizosphere soil was collected from three diseased tobacco plants in each root rot-affected plot. For the S_0 control, rhizosphere soil was collected from three healthy tobacco plants. During sampling, the entire tobacco plants were uprooted with their roots, and loose surface soil surrounding the roots was removed. Soil adhering within a 2 mm radius of the fine roots was collected and immediately cryopreserved in liquid nitrogen.

### DNA extraction, target gene amplification, and sequencing data management

Rhizosphere soil DNA was extracted using the E.Z.N.A.® Soil DNA Kit (Omega Bio-tek, D5625-02). The extracted DNA was used as the template for amplification of the V3–V4 variable regions of the bacterial 16S rRNA gene and the fungal ITS1 region. PCR products were examined using 2% agarose gel electrophoresis. Target amplicons were excised, purified, and submitted to Shanghai Majorbio Bio-pharm Technology Co., Ltd. for high-throughput sequencing using the Illumina HiSeq platform. The primer sequences are listed in Supplementary Table S1.

Illumina HiSeq paired-end reads were processed using Trimmomatic software for assembly and quality filtering. Chimeras were removed using UCHIME V4.2, and the filtered reads were assembled using FLASH software. Operational Taxonomic Units (OTUs) were clustered at a 97% similarity threshold using UPARSE software, with singleton sequences and chimeras removed during clustering. Representative OTU sequences were classified and annotated using the RDP Classifier. The Silva database was used for bacterial 16S rRNA gene annotation, and the UNITE database was used for fungal ITS annotations. Mothur software was used to calculate the OTU richness, Chao, Shannon, Simpson, and ACE indices. These metrics were used to describe the soil microbial community composition across taxonomic levels. Demultiplexed paired-end 16S rRNA gene sequencing was processed using QIIME 2.^50^ After primer sequence removal and quality filtering, sequences were denoised, merged, and screened for chimeras using the q2-dada2 plugin with default parameters.^51^ The resulting sequences were defined as amplicon sequence variants (ASVs). Taxonomy was assigned using a Naive Bayes classifier trained on the SILVA 16S rRNA database (v 138.2), with a confidence threshold of 0.70.^52^ ^,^ ^53^ Fungal ITS sequences were processed using the same ASV-inference workflow, and taxonomic assignment was performed against the UNITE fungal ITS reference database.

### Isolation and *in vitro* antagonistic activity assay

Bacteria were isolated from rhizosphere soil collected from tobacco plants mildly affected by tobacco root rot in a tobacco-growing field in Yuxi City, Yunnan Province, China. Briefly, 25 g of rhizosphere soil was suspended in sterile distilled water to a final volume of 100 mL and shaken at 28°C and 120 rpm for 30 min. The suspension was serially diluted, and 100 μL aliquots of the 10^−3^, 10^−4^ and 10^−5^ dilutions were spread onto LB agar plates. Plates were incubated at 28°C for 48 h, after which morphologically distinct colonies were selected and purified by repeated streaking on fresh LB agar plates. Purified bacterial isolates were grown in LB broth at 28°C and 200 rpm for 24 h. Cells were obtained by centrifugation at 6000 rpm for 10 min, washed, and resuspended in sterile water. Before use, the bacterial suspension was adjusted to approximately 1 × 10^8^ CFU mL^−1^. Antagonistic activity was tested against four *Fusarium* isolates associated with tobacco root rot: *F. solani* 30SL98, *F. solani* 20CJ8, *F. oxysporum* 22YJ2, and *F. oxysporum* 22JX93. The isolates were originally recovered from symptomatic tobacco roots collected in Kunming, Yunnan Province of China and were purified and identified as described previously.^54^ The identities were confirmed based on sequence analysis of the *Elongation factor* (*EF*) gene. All isolates were maintained on potato dextrose agar at 4°C and subcultured before use.

A modified dual-culture assay was adopted. A 5 mm diameter mycelial plug from the margin of a 5-d fungal colony was placed at the center of a PDA plate, and 5 μL of the bacterial suspension was spotted at a defined distance from the plug. Sterile water was used as the control. Plates were incubated at 28°C until the control colony nearly covered the plate. Colony diameters were measured, and the inhibition rate was calculated using the following formula:

$$\text{Inhibition rate}(\%)=\frac{D_{c}-D_{t}}{D_{c}}\times 100$$

where $D_{c}$ represents the colony diameter in the control, and $D_{t}$ represents the colony diameter in the bacterial treatment.

### Identification and phylogenetic analysis

Genomic DNA was extracted from the bacterial strain using the cetyltrimethylammonium bromide (CTAB) method.^55^ DNA concentration and purity were assessed using a micro-volume nucleic acid and protein analyzer. The 16S rDNA and *gyrB* fragments were amplified using specific primers, and the PCR products were sequenced by Sangon Biotech (Shanghai) Co., Ltd. The resulting sequences were compared with GenBank entries using BLAST in the NCBI database. Phylogenetic trees were constructed using MEGA 11 software (Mega, Raynham, MA, USA). The primers for amplification are listed in Supplementary Table S1.

### Evaluation of the biocontrol efficacy under pot and field conditions

The pot experiment was conducted using tobacco (*Nicotiana tabacum* L.) cultivar ‘Yunyan 87’. Tobacco seeds were sown in seedling trays containing sterilized substrate and maintained in a controlled growth room. Uniform healthy seedlings at the five-leaf stage were selected for the experiment. The pathogenic *F. oxysporum* isolate was cultured in potato dextrose broth (PDB) at 25°C and 220 rpm for 3 d. The culture was filtered through three layers of sterile gauze, and the concentration of the resulting conidial suspension was adjusted to 1 × 10^7^ conidia mL^−1^ using sterile water.

A5 was cultured in Luria-Bertani (LB) broth at 37°C and 220 rpm for 24 h. Bacterial cells were collected by centrifugation, washed and resuspended in sterile water, and the concentration was adjusted to 1 × 10^7^ CFU mL^−1^. The pot experiment consisted of three treatments: (i) a healthy control (CK), in which each pot received 20 mL of sterile water; (ii) a pathogen control (*F. oxysporum*, Fo), in which each pot received 20 mL of the Fo conidial suspension; and (iii) a biocontrol treatment (Fo + A5), in which each pot received 20 mL of the Fo conidial suspension and 20 mL of the A5 fermentation broth. To ensure that the treatments received equal liquid volumes, the CK and Fo treatments received the corresponding volume of sterile vehicle solution. Similar root-drench inoculation approaches have been used to evaluate bacterial biocontrol agents against tobacco root rot.^56^ ^,^ ^57^ The plants were maintained at 25°C under controlled greenhouse conditions. Disease symptoms were evaluated 7 days after inoculation.

The field experiment was conducted at the tobacco experimental station in Changxin, Yunlong and Dali (Yunnan Province, China) in a field with a history of naturally occurring tobacco root rot. The field had relatively uniform soil fertility, and the experiment was arranged in a randomized complete block design with three replicate plots per treatment. A5 fermentation broth containing approximately 1 × 10^7^ CFU mL^−1^ was prepared for field application. Three treatments were established: (i) CK, water control; (ii) Ba, A5 fermentation broth diluted 500-fold; (iii) a chemical fungicide control, in which 20 g of ethylicin was diluted in 30 L of water. Each plot covered 0.2 hm^2^ and was surrounded by guard rows. The treatments were applied by root drenching on the day of transplantation, with 250 mL of the corresponding solution applied per plant. All plots received identical routine agronomic management throughout the experiment. At 40 days after treatment, 20 tobacco plants were randomly selected from the central rows of each plot and assessed for root rot severity. The disease index and relative control efficacy were calculated as follows:

$$\mathrm{Disease index}=\frac{\Sigma (\mathrm{number of plants at each disease grade}\times \mathrm{corresponding grade value})}{\;\mathrm{total number of plants assessed}\times \mathrm{highest grade value}}\times 100$$

$$\mathrm{Control}\;\mathrm{efficacy}\left(\%\right)=\frac{{\mathrm{DI}}_{\mathrm{control}}-{\mathrm{DI}}_{\mathrm{treatment}}}{{\mathrm{DI}}_{\mathrm{control}}}\times 100$$

Each treatment contained three independent biological replicates (*n* = 3). Data were analyzed by one-way analysis of variance, followed by Tukey’s post hoc test at *P* < 0.05.

### RNA extraction, RNA-seq, and identification of candidate homologous genes

*F. solani* mycelia were exposed to 10% (v/v) A5 cell-free fermentation supernatant prepared by culturing A5 in LB broth, whereas mycelia cultured without A5 cell-free fermentation supernatant served as the untreated control. Samples were collected at 48 and 72 h after treatment. The experiment comprised four groups: A5 cell-free fermentation supernatant treatment at 48 h, the corresponding untreated control at 48 h, A5 cell-free fermentation supernatant treatment at 72 h, and the corresponding untreated control at 72 h. Each group contained three independent biological replicates (*n* = 3), with each replicate derived from an independently cultured fungal sample. Thus, a total of 12 biological samples were used for RNA sequencing, and one sequencing library was constructed from each biological replicate. The collected mycelia were immediately frozen in liquid nitrogen and stored at −80°C until RNA extraction. Total RNA was extracted from each biological replicate using TRIzol reagent according to the manufacturer’s instructions. The quality and integrity of the RNA were assessed using agarose gel electrophoresis and spectrophotometry. RNA samples that met the quality requirements were used for Illumina library construction and high-throughput sequencing by Suzhou Genewiz Biotechnology Co., Ltd. Adapters and low-quality sequences were removed from raw reads. The resulting clean reads were mapped to the reference transcriptome of *F. solani*. Differentially expressed genes (DEGs) were identified using $|{\log\;}_{2}\text{fold change}|\geq 1$ and false discovery rate (FDR) < 0.05 as thresholds. GO annotation was conducted using Blast2GO, and KEGG pathway enrichment analysis was performed using KOBAS.

Candidate genes associated with UDP-*N*-acetylglucosamine biosynthesis and related cellular processes were selected from the *F. solani* RNA-seq dataset according to their transcript identifiers. The predicted amino acid sequence corresponding to each transcript was retrieved from the National Center for Biotechnology Information (NCBI) protein database using the associated gene or protein accession number. Each *F. solani* protein sequence was used as a query to search the *F. oxysporum* reference proteome using the protein-sequence similarity search provided by Ensembl Fungi (https://jun2026-fungi.ensembl.org/index.html). Protein sequence similarity was assessed using BLASTP-based local alignment.^58^ ^,^ ^59^ The gene identifiers, alignment length, percentage identity, alignment score, query alignment coordinates and E value are provided in Supplementary Table S2.

### Real-time quantitative PCR (RT-qPCR) analysis

*F. oxysporum* mycelia were treated with 10% or 20% A5 cell-free culture filtrate for 48 and 72 h. Control mycelia were treated with an equal volume of sterile culture medium filtrate. After treatment, the samples were immediately frozen in liquid nitrogen and stored at −80°C until RNA extraction. Total RNA was extracted using TRIzol reagent (TaKaRa, Dalian, China) according to the manufacturer protocol. RNA concentration and purity were measured using a micro-volume spectrophotometer, and RNA integrity was assessed using agarose gel electrophoresis. First-strand cDNA was synthesized using a Vazyme reverse transcription kit (Vazyme, Nanjing, China). RT-qPCR was performed using ChamQ Universal SYBR qPCR Master Mix (Vazyme) with a two-step amplification program, followed by melting curve analysis. Three biological replicates and three technical replicates were included for each sample. The relative expression levels were calculated using the $2^{-\Delta \Delta \mathrm{Ct}}$ method, with *β-tubulin* as the internal reference gene. The RT-qPCR primers are listed in Supplementary Table S1.

### Construction and verification of deletion mutants

Deletion mutants were constructed using CRISPR/Cas9-assisted homologous recombination. For each target gene, a gene-specific sgRNA was designed within the coding sequence and ligated into the Cas9 expression plasmid after BsaI-mediated restriction digestion. The donor cassette was assembled using Gibson assembly and contained the upstream homologous arm, Hygromycin B resistance cassette, and downstream homologous arm. The donor fragment and Cas9/sgRNA plasmid were co-transformed into freshly prepared *F. oxysporum* protoplasts via PEG-mediated protoplast transformation. For each transformation, approximately 2.5 μg of Cas9/sgRNA plasmid and 2.5 μg of donor DNA were mixed with 50 μL of protoplast suspension. Following PEG/CaCl_2_-mediated transformation, protoplasts were regenerated on a selective medium containing Hygromycin B. Hygromycin-resistant transformants were transferred to PDA plates for purification. Candidate deletion mutants were first screened using diagnostic PCR. Correct target gene replacement was verified using primer pairs spanning the 5′ and 3′ recombination junctions. The loss of the target ORF was confirmed using primers located inside the deleted coding region. PCR products were sequenced when necessary to verify precise integration. The primer sequences used for mutant construction and verification are listed in Supplementary Table S1.

### Statistical analysis

All data were analyzed using GraphPad Prism 10 and are presented as the mean ± standard error. The differences between two groups were evaluated using Student’s *t*-test. Multiple comparisons were analyzed using one-way ANOVA followed by Tukey’s post hoc test. Differences were considered statistically significant at *P* < 0.05. Unless otherwise stated, all experiments included at least three biological replicates.

## Results

### Changes in the structure of the rhizosphere microbial community at different severities of tobacco root rot

The rhizosphere microbial communities were analyzed to determine how they changed during the progression of tobacco root rot. Soil samples were collected from healthy tobacco plants (S_0) and from plants showing mild (S_L), moderate (S_M), or severe (S_H) disease symptoms. Bacterial 16S rRNA gene and fungal ITS amplicons were sequenced for each sample (Figure 1). At the OTU level, the alpha diversity differed among disease severity groups for both bacterial and fungal communities. For bacteria, S_0 had lower Chao, Shannon, and ACE indices and a higher Simpson index. S_L showed the highest Chao and ACE indices and the lowest Simpson index, whereas S_M showed the highest Shannon index. Thus, bacterial richness and diversity in healthy rhizosphere soil were lower than those in some diseased soils, and bacterial richness was greatest during the mild disease stage. For fungi, S_H showed the lowest Chao, Shannon, and ACE indices and the highest Simpson index, whereas S_L showed the highest Chao, Shannon, and ACE indices. These results indicate that fungal diversity decreased in severely diseased soils, whereas mildly diseased soils retained high fungal richness and diversity.

**Figure 1.**
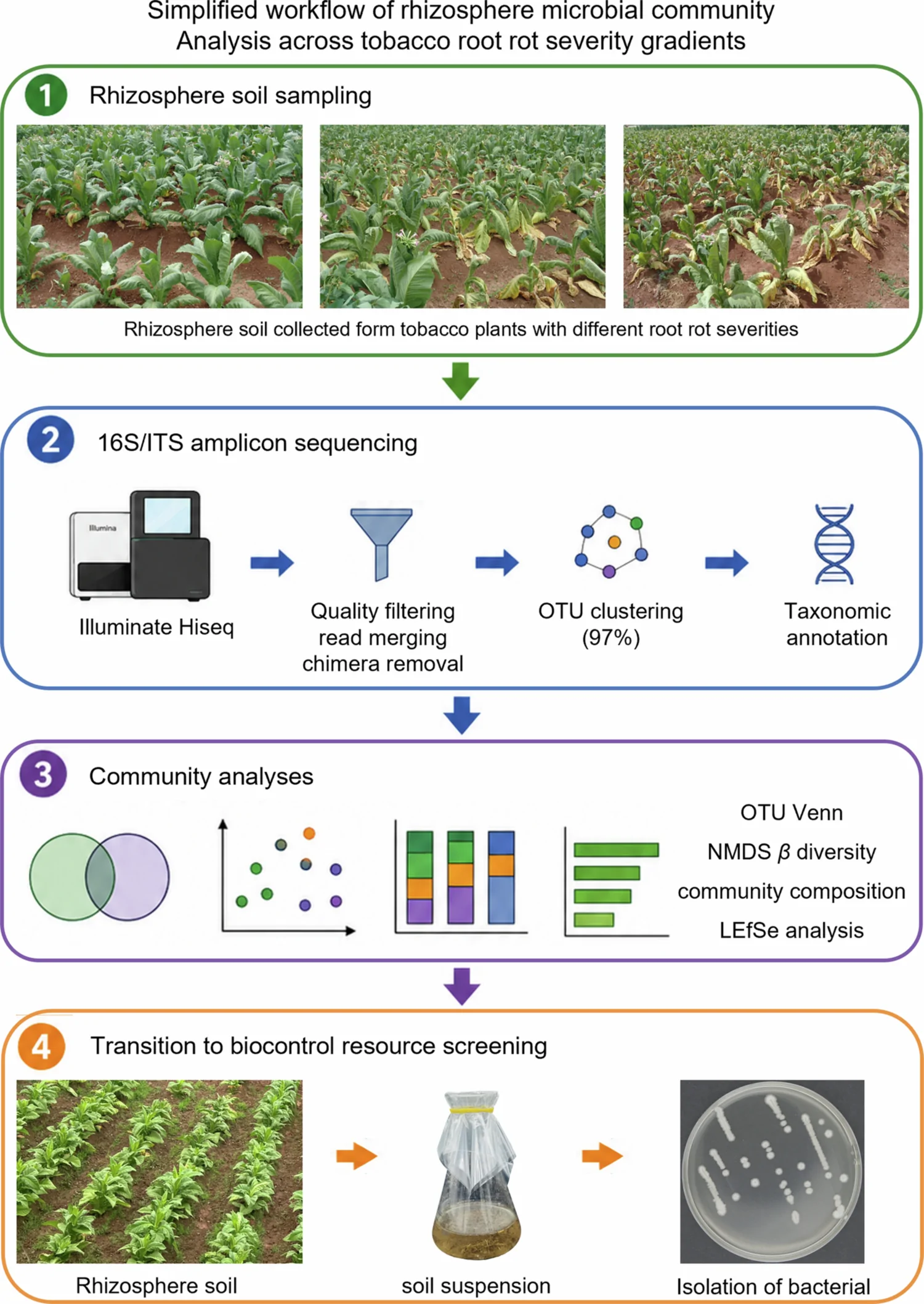
Experimental workflow for rhizosphere microbial community analysis and biocontrol resource screening.

The OTU composition indicated that tobacco root rot was associated with substantial shifts in the rhizosphere microbiome. A total of 10,402 bacterial OTUs were detected in the four soil groups. The highest number occurred in S_M (8,288), and the lowest occurred in S_0 (4,880). Among these OTUs, 3,282 were shared by all four groups, whereas 133, 508, 1,155, and 434 OTUs were unique to S_0, S_L, S_M, and S_H, respectively. In pairwise comparisons, S_L and S_M shared the most bacterial OTUs (6,151), whereas S_0 and S_H shared the fewest (4,109). This pattern indicates that severe disease was accompanied by a strong shift in bacterial community composition relative to healthy soil (Figure 2A). For fungi, 5,230 OTUs were identified in total. S_L contained the largest number of fungal OTUs (3,106), whereas S_0 contained the smallest number (1,804). The four groups shared 769 fungal OTUs, and S_0, S_L, S_M, and S_H contained 306, 856, 1,039, and 346 unique OTUs, respectively. As observed for bacteria, S_L and S_M shared the most fungal OTUs, whereas S_0 and S_H shared the fewest. These results showed that the fungal community composition was reorganized as disease severity increased (Figure 2B). In addition to the OTU-based analysis, the bacterial 16S rRNA gene and fungal ITS datasets were analyzed at the ASV level to provide a higher-resolution description of the rhizosphere microbial communities. The numbers and distributions of total, shared, and group-specific ASVs among the four disease-severity groups are summarized in Supplementary Tables S3 and S4. The OTU and ASV based taxonomic analyzes revealed broadly consistent bacterial and fungal community profiles. At the OTU level, Actinobacteriota, Proteobacteria, Chloroflexi, and Acidobacteriota were the predominant phyla across all disease-severity groups, although their relative abundances varied among groups. Similarly, the ASV analysis identified Actinomycetota (28.20%), Pseudomonadota (26.40%), Chloroflexota (15.64%), and Acidobacteriota (11.95%) as the dominant phyla, followed by Gemmatimonadota, Bacillota, and Bacteroidota (Supplementary Table S3). At the OTU level, Ascomycota was dominant across all disease-severity groups, followed by Basidiomycota and Mortierellomycota. Ascomycota was relatively more abundant in S_L and S_M, whereas Basidiomycota was more abundant in S_0 and Mortierellomycota increased in S_H. Consistently, the ASV analysis identified Ascomycota (64.80%), Basidiomycota (14.52%), and Mortierellomycota (11.64%) as the three dominant phyla (Supplementary Table S4).

**Figure 2.**
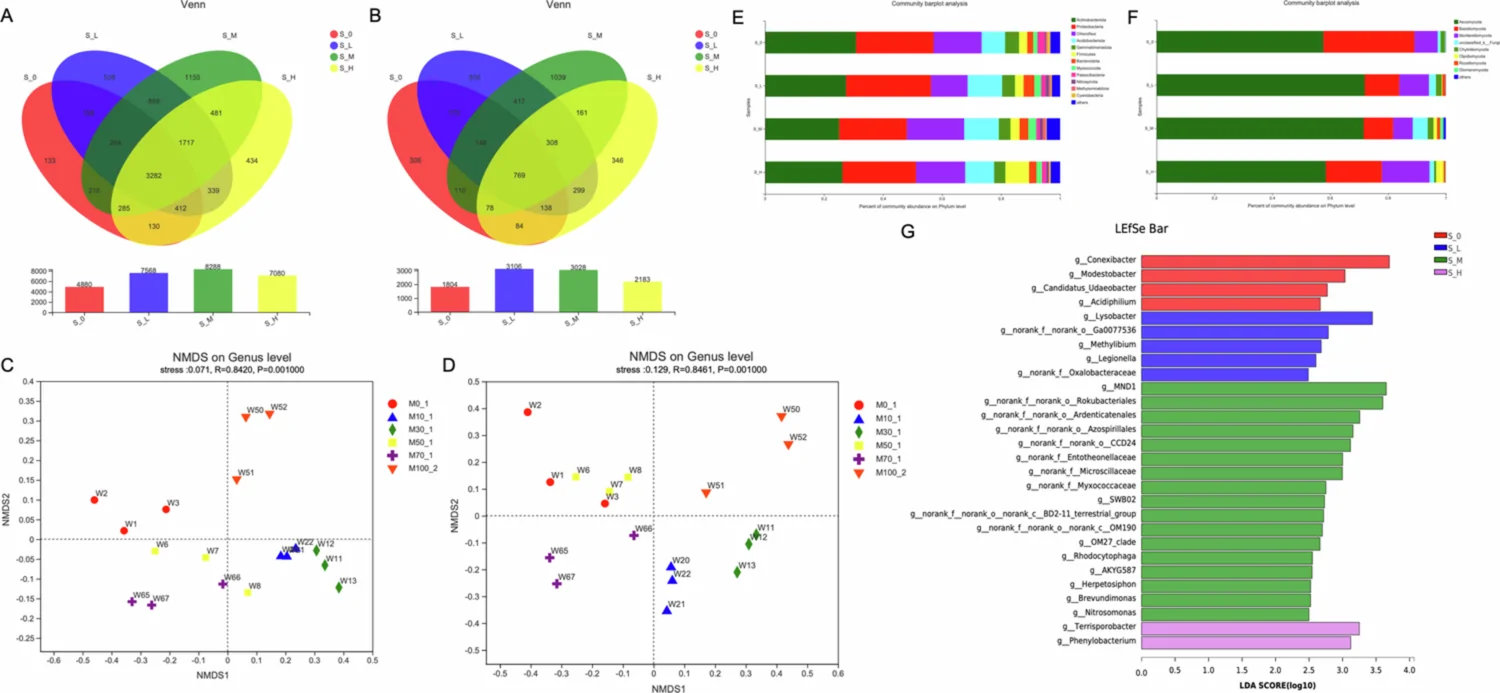
Changes in soil communities at different stages of tobacco root rot. (A) Venn diagram of bacterial OTUs. (B) Venn diagram of fungal OTUs. (C) NMDS analysis of soil bacterial community *β-*diversity based on genus-level composition. The NMDS solution had a stress value of 0.071. Significant differences among groups were detected by ANOSIM (R = 0.842, *P* = 0.001). (D) NMDS analysis of soil fungal community *β-*diversity based on genus-level composition. S_0 represents healthy plots (W1, W2, and W3), S_L represents mildly diseased plots (W20, W21, and W22), S_M represents moderately diseased plots (W6–W8 and W11–W13), and S_H represents severely diseased plots (W50–W52 and W65–W67). The NMDS solution had a stress value of 0.129. Significant differences among groups were detected by ANOSIM (R = 0.8461, *P* = 0.001). The labels M0_1, M10_1, M30_1, M50_1, M70_1, and M100_2 in the legend correspond to S_0, S_L, S_M, S_M, S_H, and S_H, respectively. (E) Analysis of bacterial community composition. (F) Analysis of fungal community composition. (G) LEfSe analysis of bacterial communities.

Genus-level *β*-diversity analysis was performed to compare overall microbial community structure among disease severity groups. In the bacterial NMDS ordination, S_H was clearly separated from S_0, S_L, and S_M, showing that severe disease markedly altered the rhizosphere bacterial community structure. The S_H samples were also relatively concentrated, suggesting that strong disease pressure may have imposed directional selection and promoted structural convergence in the bacterial community (Figure 2C). Fungal communities demonstrated a clearer disease-associated gradient. S_0 overlapped partly with S_L and S_M, but S_H was separated from the other groups. This indicates that the fungal community may remain relatively stable during the early stages of disease but undergo substantial restructuring under severe infection (Figure 2D).

At the phylum level, the dominant bacterial groups across the four soil categories were mainly Actinobacteriota, Proteobacteria, Chloroflexi, and Acidobacteriota. Compared with diseased soils, S_0 showed higher abundances of Actinobacteriota, Gemmatimonadota, Patescibacteria, and Cyanobacteria, but lower abundances of Acidobacteriota, Firmicutes, Bacteroidota, Myxococcota, and Nitrospirota (Figure 2E). Fungal communities were dominated by Ascomycota and Basidiomycota. Basidiomycota was more abundant in S_0, Mortierellomycota was enriched in S_H, and Olpidiomycota increased in relative abundance with increasing disease severity (Figure 2F). The detection frequency of *F. oxysporum* in diseased soil did not simply increase with disease severity. This observation suggests that tobacco root rot is not determined only by the relative abundance of a single pathogen but is likely shaped by the combined effects of rhizosphere microbial community structure, soil environment, and pathogen activity.

LEfSe analysis identified bacterial taxa that distinguished soils with different disease severities. At the genus level, *Conexibacter*, *Lysobacter*, *MND1*, and *Terrisporobacter* were the main discriminatory taxa among the treatment groups (Figure 2G). *Conexibacter* was significantly enriched in healthy rhizosphere soil (S_0). *Lysobacter* was enriched in mildly diseased soil (S_L), whereas MND1 and *Terrisporobacter* were the indicator taxa for S_M and S_H, respectively. As some *Lysobacter* members have been associated with antagonism against plant pathogens and disease suppression, their enrichment in S_L may reflect a microbial defense response in mildly diseased soil. In parallel, the bacterial community in healthy tobacco rhizosphere soil was distinct from that in diseased soils, and healthy rhizosphere soil under lower disease pressure may contain beneficial microbial resources. On this basis, mildly diseases tobacco rhizosphere soil was used for the subsequent isolation of culturable bacteria and screening of their antagonistic activity against tobacco root rot-associated Fusarium species.

### Isolation, identification, and biocontrol efficacy of A5 against tobacco root rot

The rhizosphere microbiome results provided a basis for screening cultivable bacteria with antagonistic potential. A total of 100 bacterial isolates from the rhizosphere of mildly diseased tobacco plants were tested for fungal pathogens. Among them, 16 isolates exhibited inhibitory activity. After rescreening, five isolates, A5, A11, A20, A46, and A63, reproducibly inhibited the growth of *F. solani* and *F. oxysporum*, with inhibition rates above 44% (Figure 3A). A5 was selected for further study because it maintained stable antagonistic activity against all four tested isolates, namely *F. solani* 30SL98, *F. solani* 20CJ8, *F. oxysporum* 22YJ2, and *F. oxysporum* 22JX93.

**Figure 3.**
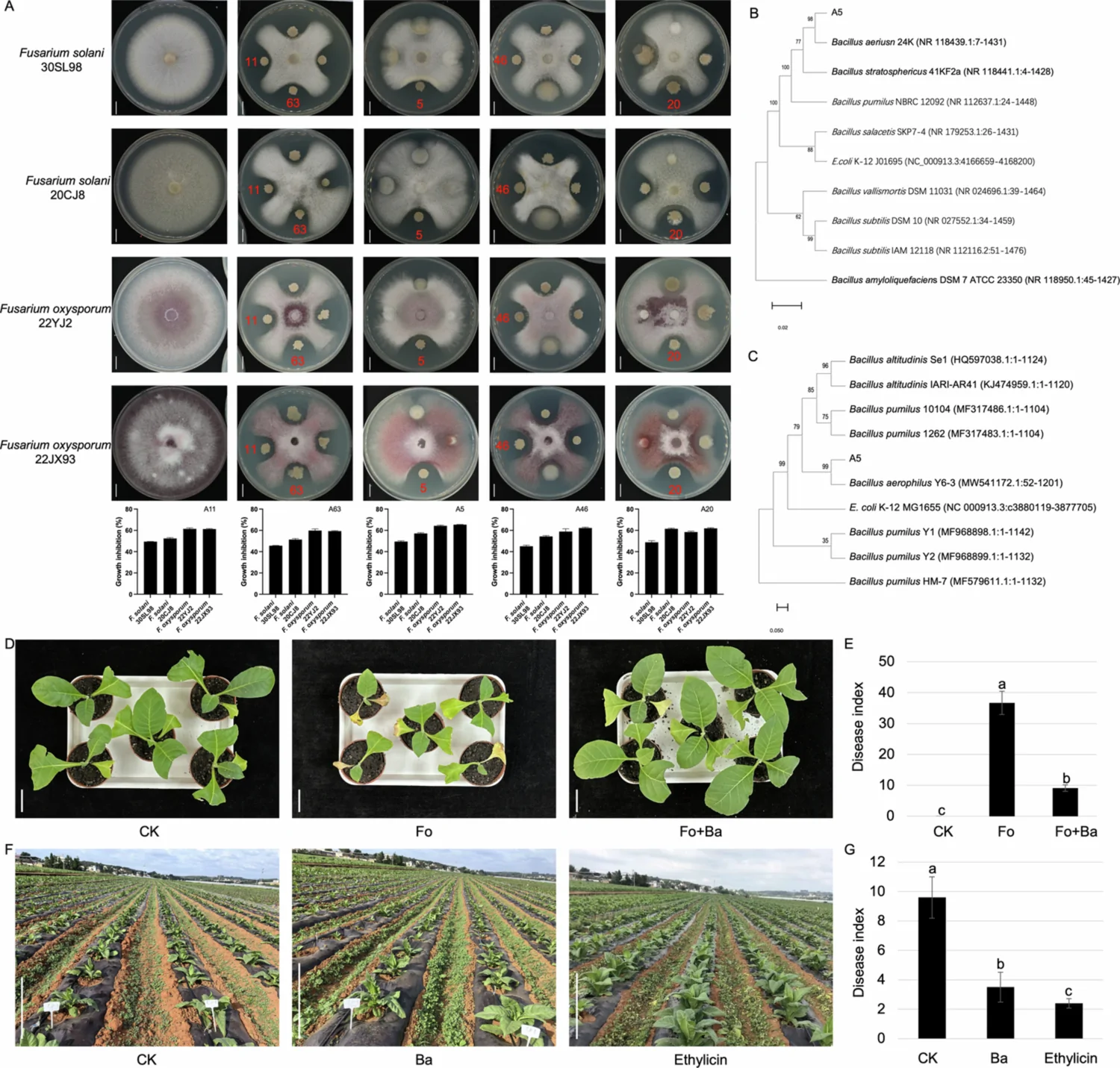
Screening, identification, and biocontrol efficacy of A5 against tobacco root rot under pot and field conditions. (A) Screening of biocontrol bacterial isolates against *Fusarium* (bar = 1 cm). (B) Phylogenetic analysis of strain A5 based on 16S rRNA gene sequences. (C) Phylogenetic analysis of strain A5 based on *gyrB* sequences. Phylogenetic trees were constructed using the neighbor-joining method with 1000 bootstrap replicates. (D) Uninoculated healthy control (CK), tobacco plants inoculated with *F. oxysporum* (Fo), tobacco plants inoculated with *F. oxysporum* and treated with strain A5 (Fo + A5) (bar = 5 cm). (E) Disease index under the different treatments at 7 d after inoculation. (F) field phenotypes of the water control, 500-fold diluted A5 fermentation broth, ethylicin treatments (bar = 1.2 m). (G) Disease indices of the field treatments. Bars represent means ± SE of three independent biological replicates (*n* = 3). Different lowercase letters indicate significant differences among treatments according to Tukey’s post hoc test at *P* < 0.05.

The taxonomic position of A5 was assessed using 16S rRNA and *gyrB* sequence analyzes. The 16S rRNA sequence was 1425 bp and shared 99.86% similarity with *Bacillus aerius* NR 118439.1. The *gyrB* sequence was 1147 bp and shared 99.39% similarity with *Bacillus aerophilus* NR 118439.1:7-1431. In phylogenetic analyzes based on both markers, A5 clustered within the *B. aerophilus* clade (Figure 3B, C). These results support the phylogenetic assignment of A5 to *B. aerophilus*.

To further evaluate the biocontrol potential of A5 in plants, tobacco seedlings inoculated with *F. oxysporum* were treated with an A5 fermentation broth under pot conditions. At 7 d after inoculation, plants with the Fo treatment exhibited pronounced symptoms of root rot compared to negative control, and the application of A5 significantly alleviated the symptoms caused by Fo (Figure 3D). Statistical data show that the disease index for the Fo treatment was 36.7, whereas it decreased to 9.1 after A5 application (Figure 3E), indicating that, under potted conditions, root application of A5 significantly alleviated Fo-induced disease symptoms. The disease-suppressive effect of A5 was evaluated in the field. The untreated control exhibited a disease index of 9.6. Application of the 500-fold-diluted A5 fermentation broth reduced the disease index to 3.5. After using ethylicin treatment, the disease index dropped to 1.5 (Figure 3F, G). These results demonstrate that the A5 fermentation broth substantially suppressed tobacco root rot under field conditions.

### Exposure to A5 cell-free fermentation supernatant is associated with extensive transcriptional reprogramming in *F. solani*

To characterize the transcriptional response of *F. solani* to A5 cell-free fermentation supernatant, fungal mycelia were exposed to 10% (v/v) A5 cell-free fermentation supernatant for 48 or 72 h and then subjected to transcriptome sequencing. The sequencing output ranged from 42.62 million to 52.54 million raw reads and from 42.55 million to 52.5 million clean reads. The clean read ratios ranged from 98.57% to 98.62%. Differential expression was analyzed separately at each time point by comparing A5 cell-free fermentation supernatant treated samples with the corresponding controls. Using |log₂(fold change)| ≥ 1 and an FDR-adjusted *P* value < 0.05 as the thresholds, 2,757 DEGs were identified at 48 h, including 1,526 upregulated and 1,231 downregulated genes. At 72 h, 3,622 DEGs were identified, of which 1,861 were upregulated and 1,761 were downregulated (Figure 4A-C). These results demonstrate that A5 cell-free fermentation supernatant was associated with extensive transcriptional changes in *F. solani*. GO (Gene Ontology) clustering analysis was conducted for all DEGs. Among the top 30 enriched GO terms, 15 were assigned to biological processes, 4 to molecular functions, and 11 to cellular components. These DEGs were mainly related to protein turnover, lipid metabolism, DNA replication-related processes, and catalytic activities. Enriched terms included proteasome-mediated protein catabolic process, fatty acid biosynthetic, regulation of DNA-dependent DNA replication initiation, heme binding, and threonine-type endopeptidase activity (Figure 4D). Collectively, these enrichment patterns indicate that the expression of genes involved in multiple cellular and metabolic processes differed following exposure to A5 cell-free fermentation supernatant. KEGG pathway enrichment analysis further showed that DEGs were associated with amino sugar and nucleotide sugar metabolism (ko00520), a pathway that includes genes involved in UDP-*N*-acetylglucosamine (UDP-GlcNAc) biosynthesis (Figure 4E). Because UDP-GlcNAc is an essential precursor for fungal chitin biosynthesis, genes associated with this metabolic route were selected for subsequent validation and functional analyzes.

**Figure 4.**
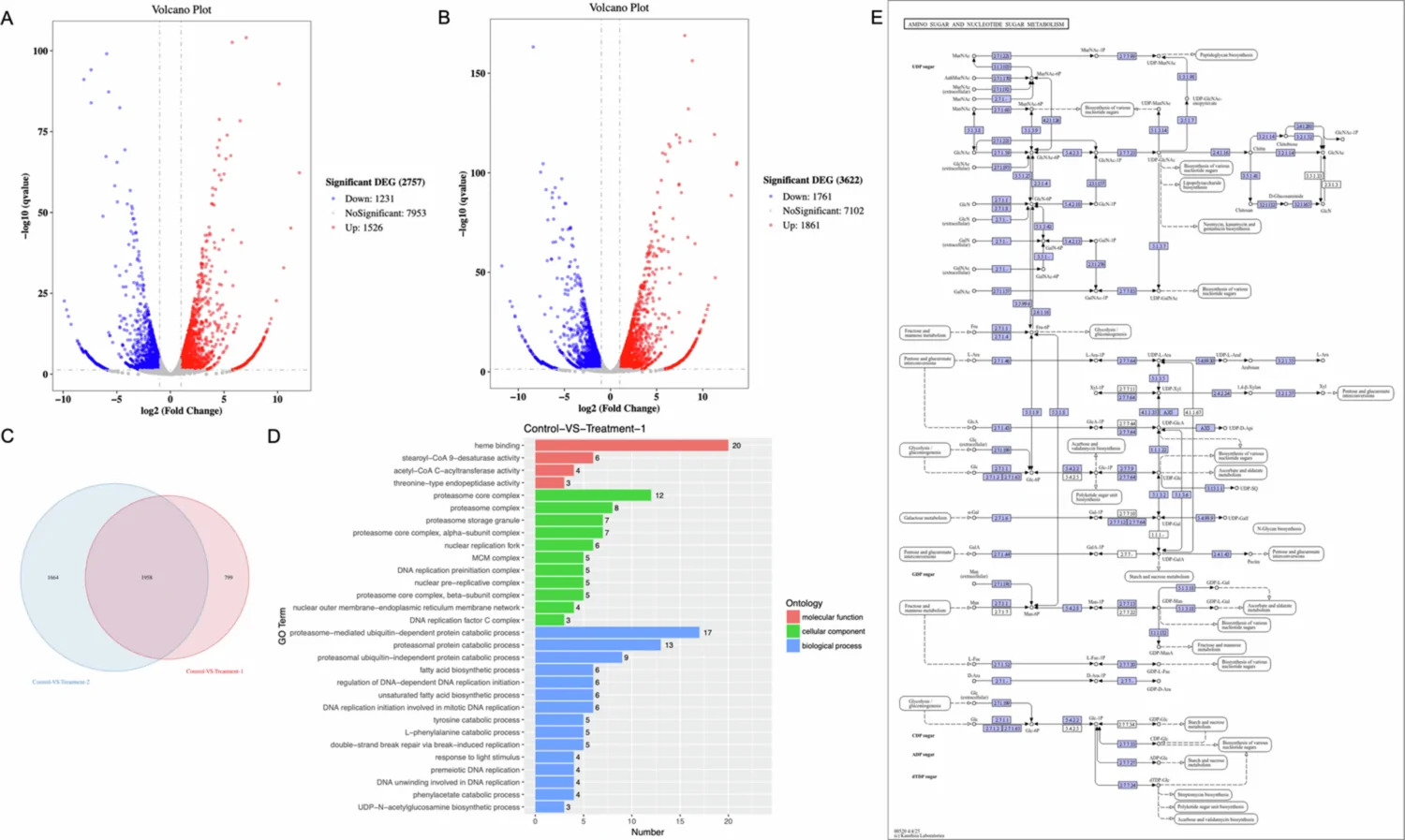
Transcriptomic responses of *F. solani* to A5 cell-free fermentation supernatant. (A) Volcano plot of DEGs in *F. solani* after 48 h of A5 supernatant treatment. (B) Volcano plot of DEGs after 72 h of treatment. (C) Venn diagram showing shared and stage-specific DEGs identified at 48 and 72 h. (D) GO enrichment analysis of DEGs in A5-treated *F. solani*. (E) Schematic representation of the UDP-*N*-acetylglucosamine biosynthesis pathway in amino sugar and nucleotide sugar metabolism.

### UDP-GlcNAc-associated candidate genes respond to A5 treatment in *F. oxysporum*

Candidate genes from the *F. solani* transcriptome were functionally evaluated in *F. oxysporum*. This species was selected because it is a major tobacco root rot-associated *Fusarium* pathogen with more complete genomic resources and a more tractable genetic transformation system. Protein sequence comparisons showed that FOXG_10443 shared 96.1% amino acid identity with MRS44_009093 over 389 amino acids. FOXG_12345 and FOXG_19023 both matched MRS44_016419, showing 91.8% identity over 546 amino acids and 87.9% identity over 149 amino acids, respectively (Supplementary Table S2). These results provide sequence-based support for selection of the three FOXG genes as candidate homologs for functional analyzes in *F. oxysporum*.

RT-qPCR was used to determine whether these candidate genes responded to A5 treatment. *F. oxysporum* mycelia were treated with 10% or 20% A5 cell-free fermentation supernatant. Under these conditions, FOXG_00190 and FOXG_00113 did not show significant changes in their expression. In contrast, FOXG_19023, FOXG_10443, and FOXG_12345 were significantly induced at 48 h, whereas their expression levels were not significantly different from those of the control at 72 h (Figure 5A). These findings identified FOXG_19023, FOXG_10443, and FOXG_12345 as early responsive candidate genes in the fungal response to A5 treatment. Deletion mutants of FOXG_19023, FOXG_10443, and FOXG_12345 were constructed using homologous recombination to assess their roles. PCR-based validation confirmed the correct target-gene replacement in the obtained mutants (Figure 5B).

**Figure 5.**
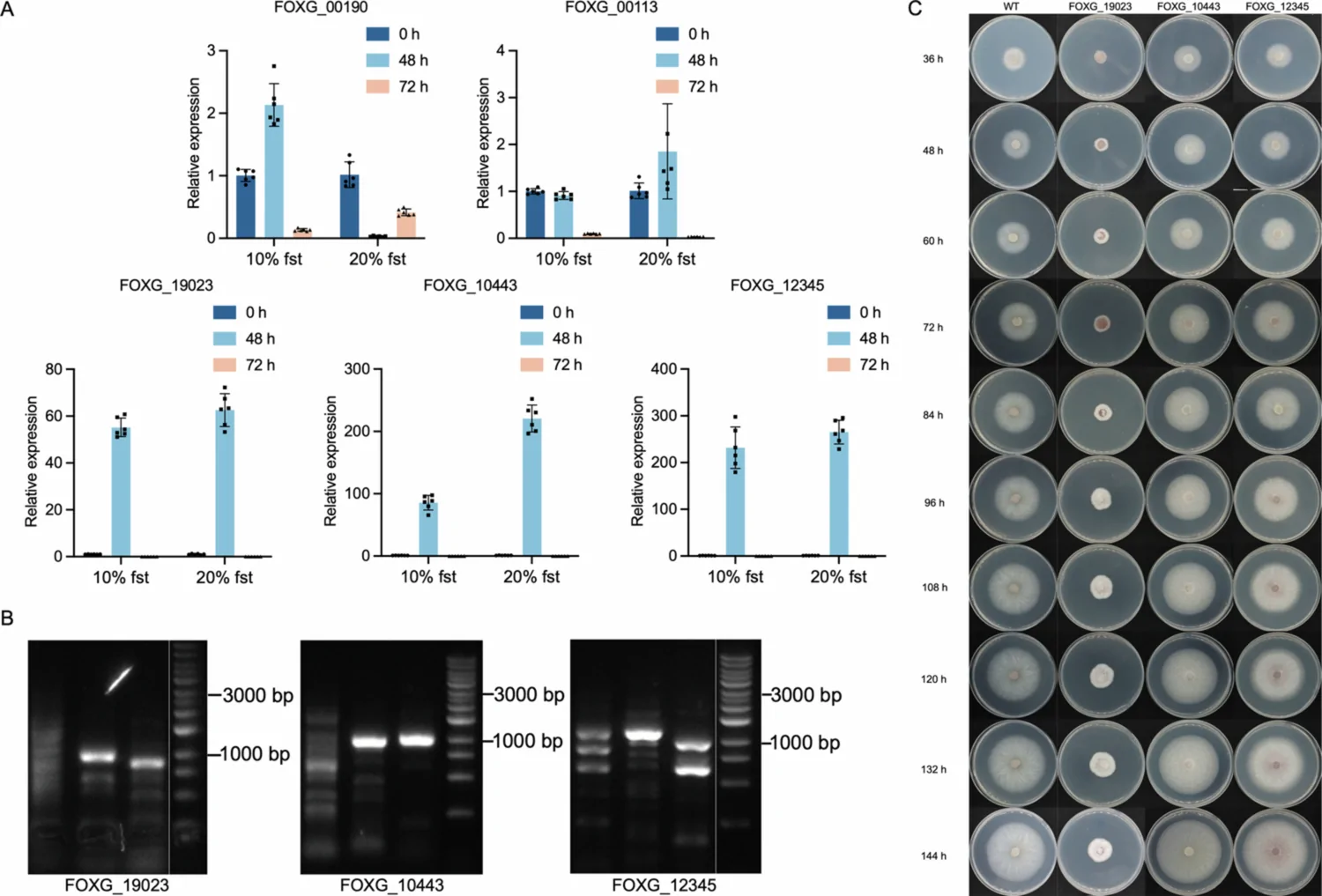
Functional validation of A5-responsive candidate genes and phenotypic characterization of deletion mutants. (A) RT-qPCR analysis of candidate gene expression in *F. oxysporum* after treatment with 10% or 20% A5 cell-free fermentation supernatant. (B) PCR-based validation of FOXG_19023, FOXG_10443, and FOXG_12345 deletion mutants. (C) Colony morphology and radial growth of the wild-type strain and deletion mutants on PDA medium.

The vegetative growth of the wild type and deletion mutants was compared on PDA medium. The FOXG_19023 deletion mutant showed the most evident growth defect among the three mutants, with a significantly reduced hyphal diameter during cultivation compared with the wild type (Figure 5C). The FOXG_10443 and FOXG_12345 mutants exhibited weaker growth defects under the same conditions. These results suggest that FOXG_19023 contributes to normal vegetative growth in *F. oxysporum* and represents a candidate gene for further investigation of the fungal response to A5 treatment.

## Discussion

*Bacillus*-based biopesticides are promising alternatives to chemical fungicides because they combine broad antagonistic activity with multiple modes of action, including the production of cyclic lipopeptides, polyketides, antimicrobial peptides, volatile compounds, and hydrolytic enzymes, as well as niche competition, biofilm formation, and induction of host resistance.^19,20,35,60–64^ In this study, A5, isolated from the rhizosphere of mildly diseased tobacco, antagonized four tobacco root rot-associated *Fusarium* isolates and was identified as *Bacillus aerophilus* based on *gyrB* phylogeny (Figure 3A, C). Importantly, its activity extended beyond dual-culture assays: A5 reduced the disease index in both Fo-inoculated pot experiments and naturally infested fields (Figure 3D-G). Similar pot and field suppression has been reported for *Bacillus amyloliquefaciens* HN11 and a *Bacillus*-based synthetic community,^56^ ^,^ ^57^ supporting the practical potential of *Bacillus* strains against tobacco *Fusarium* root rot. The lower efficacy commonly observed under field conditions probably reflects variation in soil properties, indigenous microbial competition, environmental conditions, rhizosphere colonization, and pathogen pressure.^56^ ^,^ ^57^ Nevertheless, the field performance of A5 indicates that it is a promising candidate for further formulation optimization and multi-location evaluation.

The compounds responsible for the antifungal activity of A5 remain to be identified. *Bacillus* species can produce surfactins, iturins, fengycins, polyketides, antimicrobial peptides, volatile compounds, and extracellular enzymes,^35^ ^,^ ^65^ ^,^ ^66^ and combinations of lipopeptides may act synergistically against fungi.^67^ Activity of A5 may result from one predominant compound or the combined action of several extracellular factors. Genome mining, bioassay-guided fractionation, and LC-MS/MS analysis will be useful for identifying the principal active compounds and linking them to antifungal activity.

Exposure to A5 cell-free fermentation supernatant induced extensive transcriptional reprogramming in *F. solani*, with 2,757 and 3,622 differentially expressed genes at 48 and 72 h, respectively (Figure 4A-C). Enriched functions included not only UDP-*N*-acetylglucosamine-associated processes but also protein turnover, lipid biosynthesis, DNA replication, and catalytic activities (Figure 4D, E). The breadth of this response suggests that A5 cell-free fermentation supernatant does not affect an isolated pathway alone; it may provoke a coordinated fungal stress response involving growth regulation, redox balance, membrane homeostasis, and cellular repair. Fungal exposure to environmental, oxidative, nutritional, and chemical stresses can similarly alter large transcriptional programs,^68^ ^,^ ^69^ while some *Bacillus* lipopeptides disrupt fungal membranes and consequently disturb ion homeostasis, oxidative balance, organelle function, and gene expression.^70^ Thus, the large number of DEGs is compatible with broad cellular stress, but DEG numbers alone cannot distinguish targeted inhibition from cytotoxicity. Broad-spectrum inhibition denotes activity against multiple fungal taxa, whereas cytotoxicity requires direct evidence of extensive or irreversible cellular injury. A primary target-associated response would be supported by early changes at subinhibitory concentrations preceding loss of viability, whereas membrane leakage, ROS accumulation, mitochondrial dysfunction, and failure to regrow would favor generalized cellular damage. Antifungal agents may also initiate effects at a preferred cellular site and subsequently generate widespread secondary stress responses.^71^ Accordingly, the UDP-GlcNAc-related enrichment is best interpreted as a candidate responsive process within a broader antifungal response rather than as proof of a unique primary target. Because RNA-seq used untreated mycelia rather than a medium-matched control containing 10% uninoculated LB broth, possible contributions of medium components cannot be excluded. The transcriptional changes are therefore interpreted as responses to the complete A5 cell-free fermentation supernatant, and an identically processed uninoculated-medium control should be included in future studies.

Functional validation was performed in *F. oxysporum* because it has more complete genome annotation and a more established genetic transformation system than *F. solani.* ^72^ This strategy is biologically supported by the conservation of UDP-GlcNAc biosynthesis and chitin production in filamentous fungi.^47^ ^,^ ^73^ ^,^ ^74^ Homology analysis further linked the selected *F. solani* candidates to their *F. oxysporum* counterparts, allowing the transcriptome to serve as a discovery dataset for RT-qPCR and knockout experiments. Nevertheless, conservation does not necessarily imply identical regulation or function between species; therefore, this cross-species analysis supports the relevance of the candidate pathway but does not substitute completely for direct genetic validation in *F. solani*.

The fungal cell wall provides a plausible framework for interpreting the transcriptional and phenotypic responses. Chitin and *β*-glucans form the conserved inner wall skeleton and are essential for hyphal morphology, turgor resistance, stress adaptation, and host invasion.^43^ ^,^ ^75^ ^,^ ^76^ UDP-GlcNAc is the direct sugar donor for chitin synthesis, and chitin synthases catalyze the transfer GlcNAc from UDP-GlcNAc to the growing chitin chain.^47^ ^,^ ^77^ The essential role and conserved architecture of this pathway make chitin biosynthesis an important framework for antifungal intervention.^47,49,78–80^ In this study, A5 cell-free fermentation supernatant affected, at the transcriptional level, genes associated with UDP-GlcNAc-dependent cell wall precursor processes. RT-qPCR showed that FOXG_19023, FOXG_10443, and FOXG_12345 were induced by A5 cell-free fermentation supernatant (Figure 5A). Among the corresponding deletion mutants, the FOXG_19023 mutant exhibited the strongest growth inhibition phenotype (Figure 5B, C), supporting an important role for this gene in normal hyphal growth. This finding is consistent with evidence that disruption of UDP-GlcNAc-associated processes impairs fungal growth and cell wall integrity. For example, silencing *SsAGM1* in *Sclerotinia sclerotiorum* reduced UDP-GlcNAc and chitin contents and compromised vegetative growth and pathogenicity.^80^ FOXG_19023 may therefore function in an essential or compensatory response that helps maintain growth under cell wall-associated stress.

The present evidence does not yet establish the biochemical function of FOXG_19023 or confirm UDP-GlcNAc metabolism as the exclusive target of A5. Direct measurements of UDP-GlcNAc and chitin, cell wall integrity assays, mutant complementation, and analysis using purified A5 compounds would refine this mechanistic model.

Taken together, this study identifies A5 as a promising biocontrol bacterium against tobacco root rot-associated *Fusarium*. These findings connect the disease-suppressive performance of A5 with a biologically relevant fungal response framework and provide candidate genes and pathways for elucidating *Bacillus-Fusarium* interactions and developing A5-based strategies for sustainable management of tobacco root rot.

## Supplementary Material

Supplementary Material Supplementary Table.xlsx

## Data Availability

All data supporting the findings of this study are available within the paper and its supplementary information.
